# Insm1a Regulates Motor Neuron Development in Zebrafish

**DOI:** 10.3389/fnmol.2017.00274

**Published:** 2017-08-28

**Authors:** Jie Gong, Xin Wang, Chenwen Zhu, Xiaohua Dong, Qinxin Zhang, Xiaoning Wang, Xuchu Duan, Fuping Qian, Yunwei Shi, Yu Gao, Qingshun Zhao, Renjie Chai, Dong Liu

**Affiliations:** ^1^School of Life Science, Nantong University Nantong, China; ^2^Co-innovation Center of Neuroregeneration, Key Laboratory of Neuroregeneration of Jiangsu and Ministry of Education, Nantong University Nantong, China; ^3^MOE Key Laboratory of Model Animal for Disease Study, Model Animal Research Center, Nanjing University Nanjing, China; ^4^Key Laboratory for Developmental Genes and Human Disease, Ministry of Education, Institute of Life Sciences, Southeast University Nanjing, China

**Keywords:** *insm1a*, motor neuron, differentiation, zebrafish, development

## Abstract

Insulinoma-associated1a (insm1a) is a zinc-finger transcription factor playing a series of functions in cell formation and differentiation of vertebrate central and peripheral nervous systems and neuroendocrine system. However, its roles on the development of motor neuron have still remained uncovered. Here, we provided evidences that *insm1a* was a vital regulator of motor neuron development, and provided a mechanistic understanding of how it contributes to this process. Firstly, we showed the localization of *insm1a* in spinal cord, and primary motor neurons (PMNs) of zebrafish embryos by *in situ* hybridization, and imaging analysis of transgenic reporter line *Tg(insm1a: mCherry)*^*ntu805*^. Then we demonstrated that the deficiency of *insm1a* in zebrafish larvae lead to the defects of PMNs development, including the reduction of caudal primary motor neurons (CaP), and middle primary motor neurons (MiP), the excessive branching of motor axons, and the disorganized distance between adjacent CaPs. Additionally, knockout of *insm1* impaired motor neuron differentiation in the spinal cord. Locomotion analysis showed that swimming activity was significantly reduced in the *insm1a*-null zebrafish. Furthermore, we showed that the *insm1a* loss of function significantly decreased the transcript levels of both *olig2* and *nkx6.1*. Microinjection of *olig2* and *nkx6.1* mRNA rescued the motor neuron defects in *insm1a* deficient embryos. Taken together, these data indicated that *insm1a* regulated the motor neuron development, at least in part, through modulation of the expressions of *olig2* and *nkx6.1*.

## Introduction

In vertebrates, motor neurons have precise subtype identities that characterized by a number of morphological criteria, such as soma location, and shape, axon path, and target muscle innervation (Shirasaki and Pfaff, 2002; Lewis and Eisen, 2003). Meanwhile, motor neurons generally extend their axonal trajectory with a highly stereotyped manner during the nervous system development (Eisen, 1991; Palaisa and Granato, 2007). It has been reported that in chick and bullfrog, their motor neuron axons always followed the conservative pathways in order to project to appropriate regions of target musculatures (Landmesser, 1980; Farel and Bemelmans, 1985). In the embryo and larva of zebrafish, there are two different kinds of spinal motor neurons, which are called primary motor neurons (PMNs), and secondary motor neuron (SMNs) (Myers, 1985; Myers et al., 1986). The PMNs can be further classified into three groups, caudal primary motor neurons (CaP), middle primary motor neurons (MiP), and rostral primary motor neurons (RoP), by the positions of somata in the spinal cord, and the trajectory of neuron axons (Myers et al., 1986; Westerfield et al., 1986). CaPs, whose somata locate in the middle of each spinal cord hemisegment, can innervate ventral axial muscle, and have been well-studied because of their easy observation and distinct axon projection (Myers et al., 1986; Rodino-Klapac and Beattie, 2004). MiPs project axons to innervate the dorsal axial muscle, while RoPs project axons to control the middle muscle (Rodino-Klapac and Beattie, 2004). Although the somata of the three identifiable PMNs are localized in different position in the spinal cord, their axons pioneer to the myoseptum through a mutual exit point (Eisen et al., 1986). Due to the identifiability of the three kinds of PMNs, they have already become an excellent system to study motor axon guidance and their intraspinal navigation (Beattie et al., 2002).

The insulinoma-associated 1 (*insm1*) gene, which is first isolated from an subtraction cDNA library of insulinoma tumor cells, encodes a DNA-binding zinc finger transcription factor with SNAG repressor motifs in N-terminal as well as Cys2-His2 Zn finger motifs in C-terminal, and widely expresses in the developing nervous system, endocrine cells, pancreatic cells, and related neuroendocrine tumor cells (Goto et al., 1992; Xie et al., 2002; Jacob et al., 2009; Lan and Breslin, 2009; Jia et al., 2015b). Consequently, extensive studies focused on the biological function of *insm1* in nervous, and endocrine cell proliferation, differentiation, and transformation have been reported in the model organisms (Farkas et al., 2008; Wildner et al., 2008; Lan and Breslin, 2009; Ramachandran et al., 2012; Jia et al., 2015a,b). For example, in the *insm1* knockout mice, its endocrine progenitor in the developing pancreas were less differentiated, meanwhile hormone production, and cell migration also exhibited seriously defects (Osipovich et al., 2014). Farkas et al. reported that compared to the wild type and heterozygous mice, the number of basal progenitors in the *insm1* null dorsal telencephalon (dTel) was decreased almost half, and the radial thickness of dTel cortical plate as well as the neurogenesis in the neocortex were also predominantly reduced after lacking *insm1* gene (Farkas et al., 2008). In the zebrafish, *insm1a* can regulate a series of related genes, which are necessary for the Müller glia (MG) formation, and differentiation as well as the zone definition of injury-responsive MG to participate in the retina regeneration (Ramachandran et al., 2012). Moreover, it was also reported that during the development of zebrafish retina, *insm1* could regulate cell cycle kinetics and differentiation of the progenitor cells by acting the upstream of the basic helix-loop-helix (bHLH) transcription factors, and the photoreceptor specification genes (Forbes-Osborne et al., 2013). Although *insm1a* is widely detected in the nervous system and its necessity in the brain and retina development have been also illuminated well, little is known about the function and molecular mechanisms of *insm1a* on the formation and development of other neuronal types, especially in the zebrafish.

The zebrafish has become an excellent model system to investigate the mechanisms of the neuron formation and its axonal pathfinding due to the accessible observation of motor neurons from the initial stages of embryo development (Zelenchuk and Bruses, 2011). Here, we examined the function of *insm1a* in the primary motor neurons development by CRISPR/ Cas9-mediated knockout in the *Tg(mnx1:GFP)*^*ml*2^ transgenic zebrafish and investigated the possible transcriptional network during this process.

## Materials and methods

### Zebrafish line and breeding

The zebrafish embryos and adults were maintained in zebrafish Center of Nantong University under conditions in accordance with our previous protocols (Xu et al., 2014; Wang et al., 2016). The transgenic zebrafish line, *Tg(mnx1:GFP)*^*ml*2^, has been described in the previous work (Zelenchuk and Bruses, 2011).

### RNA isolation, reverse transcription and quantitative PCR

Total RNA was extracted from zebrafish embryos by TRIzol reagent according to the manufacturer's instructions (Invitrogen, USA). Genomic contaminations were removed by DNaseI, and then 2 μg total RNA was reversely transcribed using a reversed first strand cDNA synthesis kit (Fermentas, USA) and stored at −20°C. qRT-PCR was performed using the corresponding primers (Supplementary Table 1) in a 20 μl reaction volume with 10 μl SYBR premix (Takara, Japan) and *elongation factor 1a* (*ef1a*) was used as the internal control. All samples were analyzed in triplicate.

### Whole mount *In situ* hybridization

A 501 bp cDNA fragment of *insm1a* was amplified from the cDNA library that established from wild type (WT) AB embryos using the specific primers of *insm1a* F1 and *insm1a* R1 (Supplementary Table 1). Digoxigenin-labeled sense and antisense probes were synthesized using linearized pGEM-T-easy vector subcloned with this *insm1a* fragment by *in vitro* transcription with DIG-RNA labeling Kit (Roche, Switzerland). Zebrafish embryos and larvae were collected and fixed with 4% paraformaldehyde (PFA) in phosphate-buffered saline (PBS) for one night. The fixed samples were then dehydrated through a series of increasing concentrations of methanol and stored at −20°C in 100% methanol eventually. Whole mount *in situ* hybridization was subsequently performed as described in the previous study (Huang et al., 2013).

### Establishment of *Tg(insm1a: EGFP)*^*ntu804*^ and *Tg(insm1a: mCherry)^*ntu805*^* transgenic line

Transgenic zebrafish were created using the Tol2kit transgenesis system and Gateway vectors. The *insm1a* promoter was cloned and insert into the p5E-MCS entry vector. A multiSite Gateway vector construction reaction (Invitrogen, USA) was conducted with the resulting p5E-insm1a together with pME-EGFP (or mCherry) and p3E-polyA subcloned into the pDestTol2pA2 to produce *insm1a: EGFP* or *insm1a: mCherry* construct. Subsequently, this construct was co-injected with *tol2-transposase* mRNAs into zebrafish one to two-cell-stage embryos to create the *Tg(insm1a: EGFP)*^*ntu804*^ and *Tg(insm1a: mCherry)*^*ntu805*^ transgenic line.

### sgRNA/ Cas9 mRNA synthesis and injection

Cas9 mRNA was obtained by *in vitro* transcription with the linearized plasmid pXT7-Cas9 according to the procedure previously described. The sgRNAs were transcribed from the DNA templates that amplified by PCR with a pT7 plasmid as the template, a specific forward primer and a universal reverse primer (Supplementary Table 1) (Chang et al., 2013; Qi et al., 2016). Transgenic zebrafish lines *Tg(mnx1:GFP)*^*ml*2^, were natural mated to obtain embryos for microinjection. One to two-cell stage zebrafish embryos were injected with 2–3 nl of a solution containing 250 ng/μl Cas9 mRNA and 15 ng/μl sgRNA. At 24 h post fertilization (hpf), zebrafish embryos were randomly sampled for genomic DNA extraction according to the previous methods to determine the indel mutations by sequencing.

### Morpholino and mRNAs injections

Translation blocking antisense Morpholino (MOs; Gene Tools) against the ATG-containing sequence was designed (5′-AAATCCTCTGGGCATCTTCGCCAGC-3′) to target the translation start site according to the manufacturer's instruction and the other MO oligo (5′-CCTCTTACCTCAGTTACAATTTATA-3′) was used as standard control. The MOs were diluted to 0.3 mM with RNase-free water and injected into the yolk of one to two-cell stage embryos and then raised in E3 medium at 28.5°C.

The cDNAs containing the open reading frame of the target genes were cloned into PCS2^+^ vector respectively and then were transcribed *in vitro* using the mMESSAGE mMACHIN Kit (Ambion, USA) after the recombinant plasmids linearized with NotI Restriction Enzyme (NEB, England), and then the capped mRNAs were purified by RNeasy Mini Kit (Qiagen, Germany). 2 nl target genes and *mCherry* mRNA mixture (1:1) were injected at 20 ng/μl into 1/2-cell stage embryos.

### Locomotion analysis in zebrafish larvae

To determine whether the deficiency of *insm1a* affect spontaneous movement, knockout, and normal larvae were raised in a 24-well-culture plate with one larva in each well-filled with 1 ml E3 medium. The 24-well-culture plate was transferred to the Zebralab Video-Track system (Zebrabox, France) equipped with a sealed opaque plastic box that kept insulated from laboratory environment, an infrared filter and a monochrome camera. After adapting for 30 min, traveled distances of the larvae were videotaped with every 5 mins forming a movement distance and trajectory by the linked software.

### Microscopy and statistical analysis

Zebrafish embryos were anesthetized with E3/0.16 mg/mL tricaine/1% 1-phenyl-2-thiourea (Sigma, USA) and embedded in 0.8% low melt agarose, and then were examined with a Leica TCS-SP5 LSM confocal imaging system. For the results of *in situ* hybridization, Photographs were taken using an Olympus stereomicroscope MVX10. Statistical comparisons of the data were carried out by student's *t*-test or one-way analysis of variance (ANOVA) followed by Duncan's test, and *P* < 0.05 were considered statistically significant. All statistical analysis was performed using the SPSS 13.0 software (SPSS, USA).

## Result

### *Insm1a* is expressed in spinal cord and PMNs of zebrafish

**Figure 1:**
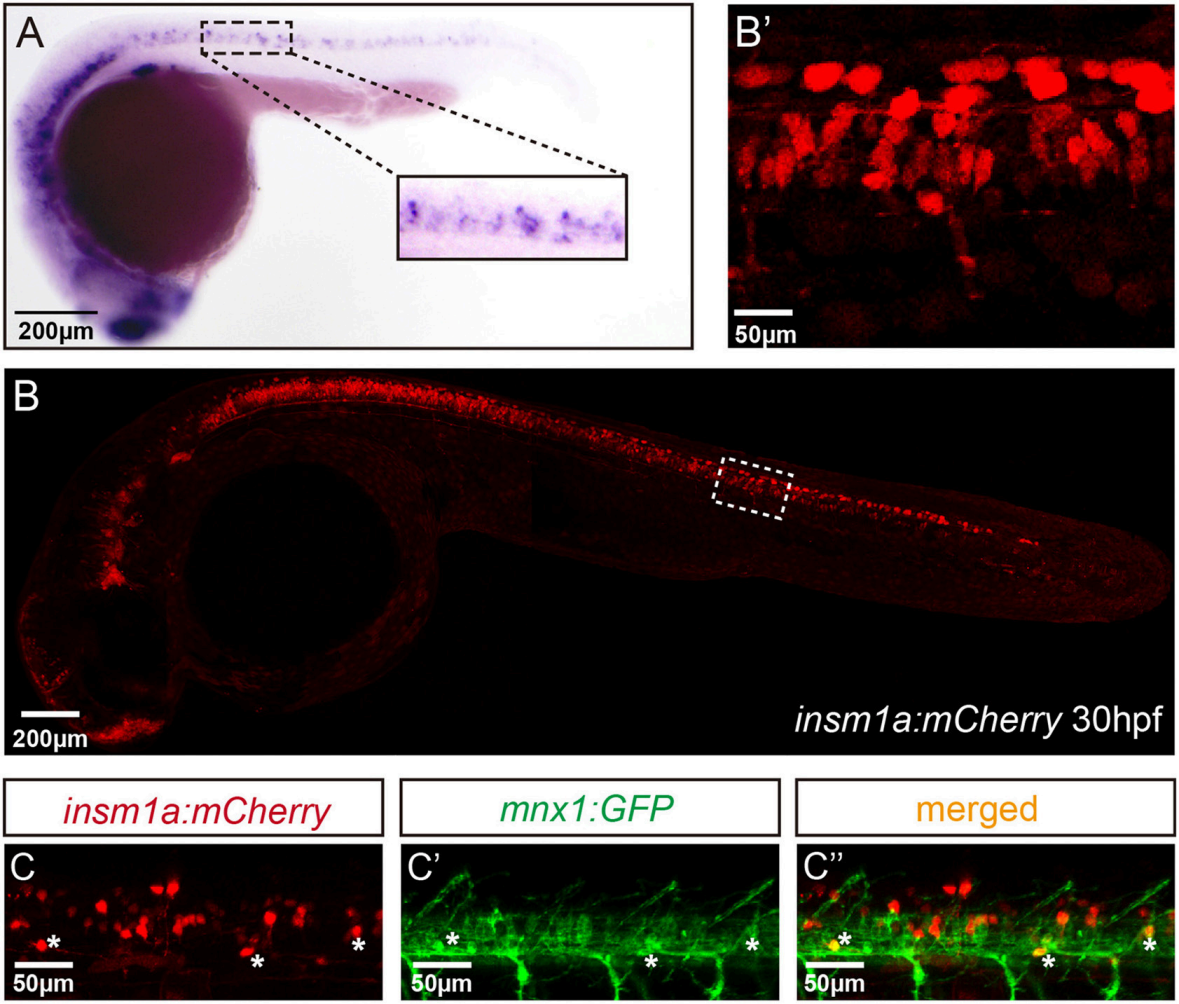
*Insm1a* expression in embryonic zebrafish spinal cord and primary motor neurons. **(A)** At 24 hpf, the *in situ* hybridization signal of insm1a is localized in the spinal cord., Scale bar = 200 μm. **(B)** The confocal imaging analysis of the transgene *insm1a:mCherry* expression at 30 hpf. Square in dash line indicates the magnified region in **(B')** Scale bar = 50 μm. **(C,C',C”)**. Confocal imaging analysis of *Tg(mnx1:GFP)*^*ml*2^ × *Tg(insm1a: mCherry)*^*ntu805*^ transgenic line.

To analyze the expression of *insm1a* in zebrafish nervous system, we performed the whole amount *in situ* hybridization (WISH) analysis with a digoxigenin-labeled *insm1a* probe. Similar to the previous study (Lukowski et al., 2006), at late somitogenesis (24 hpf) *insm1a* transcripts were apparently localized in ventral part of the neurons in the spinal cord, where most of the motor neurons located at this stage (Figure 1A).

To further determine the localization of *insm1a*, we generated the *Tg(insm1a: EGFP)*^*ntu804*^ and *Tg(insm1a: mCherry)*^*ntu805*^ transgenic zebrafish lines, in which the *insm1a* promoter directed expression of EGFP or mCherry respectively. It was shown that at 30 hpf the *insm1a:mCherry* and *insm1a:EGFP* expression was observed in the spinal cord, retina and brain, which was similar with the results of *in situ* hybridization (Figures 1B,B'; Supplementary Figures 1A,A') (Lukowski et al., 2006). In addition, we found that *insm1a:EGFP* expression was highly activated in Müller glia of injury sites in adult zebrafish retina (Supplementary Figure 1B), which was consistent with the ISH data carried out by other researchers (Ramachandran et al., 2012). These results suggested that the transgenes recapitulated the endogenous *insm1a* expression.

To investigate whether *insm1a* is expressed in motor neurons, we outcrossed *Tg(insm1a: mCherry)*^*ntu805*^ transgenic line with *Tg(mnx1:GFP)*^*ml*2^ line, in which motor neurons were labeled with GFP (Zelenchuk and Bruses, 2011). We found that the GFP^+^ motor neurons were also labeled with mCherry fluorescence (Figures 1C–C”), suggesting *insm1a* was expressed in motor neurons.

### Knockout of *insm1a* caused primary motor neurons developmental defect

In order to examine whether *insm1a* is required for the development of motor neuron, the CRISPR/Cas9 system was utilized to knockout *insm1a* in *Tg(mnx1:GFP)*^*ml*2^ transgenic zebrafish line. To ensure complete disruption of functional proteins, we chose the target sites near the translation start codon (ATG) in the exon1 of zebrafish *insm1a* (Supplementary Figure 2A). The selected gRNA-Cas9 system efficiently induced mutations in the targeting site with four types of mutations were identified (Supplementary Figure 2B). The mutated alleles included a 5-bp deletion, an 8-bp deletion and two 10-bp deletions, which all resulted in reading frame shift and premature translation termination (Supplementary Figure 2C). In addition, these lines showed the same phenotypes and the 8-bp deletion mutant line was used for the following experiments.

**Figure 2:**
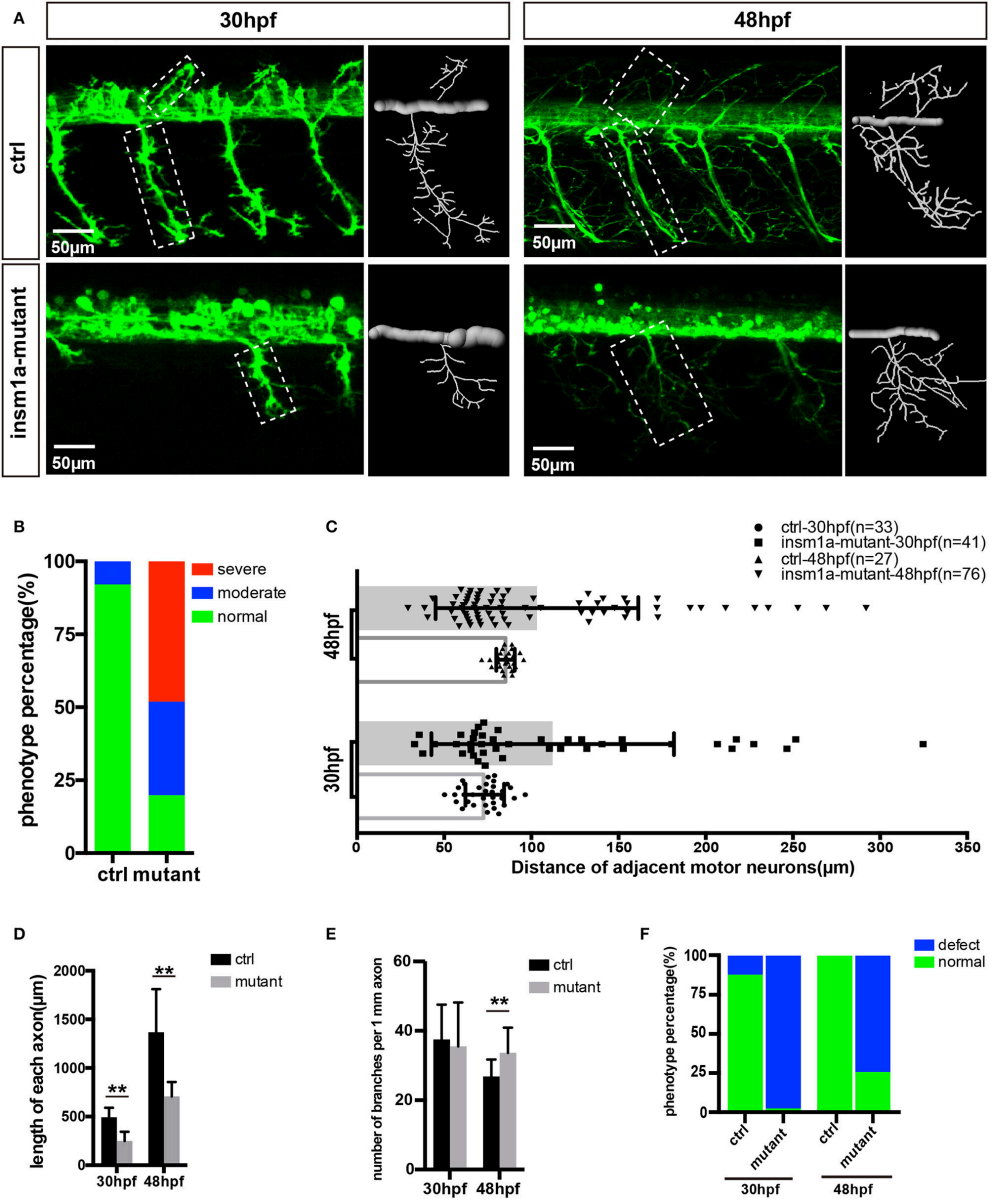
Primary motor neuron morphogenesis defects in the *insm1a* mutant zebrafish embryos. **(A)** Confocal imaging analysis of primary motor neuron in control group and *insm1a* mutant groups at 30 hpf and 48 hpf *Tg(mnx1:GFP)*^*ml*2^. Caps in dash line are showed in diagrams. Scale bar = 50 μm. **(B)** Quantification of zebrafish embryos with abnormal Caps. The zebrafish embryos are classified into three categories according to its loss degree: severe group with over 80% loss of Cap primary motor neuron, moderate group with <80% loss, and normal group with <20% loss. **(C)** Quantification of distance between adjacent motor neurons (μm) in control group and *insm1a* mutant groups at 30 hpf (*n* = 33 and 41 respectively) and 48 hpf (*n* = 27 and 76 respectively). **(D,E)** The length and branching number of Cap axons in control group and *insm1a* mutant groups at 30 and 48 hpf. Asterisks above the bars indicate significant differences (^**^P < 0.01). **(F)** Quantification of zebrafish embryos with abnormal Caps at 30 and 48 hpf.

It was observed that *insm1a* knockout caused obvious developmental defect of motor neurons (Figure 2). Firstly, the number of MiPs and CaPs were significantly reduced in the *insm1a* mutants (Figure 2A). We counted the number of Caps and classified the zebrafish embryo into three categories by its defective degree: severe group with over 80% loss of Caps, moderate group with < 80% loss and normal group with <20% loss (In the following statistical analysis, the zebrafish with <20% loss was defined as normal, whereas, it was abnormal). These results revealed that 48.1% severe and 32.1% moderate defect were found in the *insm1a* mutants, while there was only 7.9% moderate defect in the control group (Figure 2B). Similarly, the MiPs were also obviously impaired in *insm1a* knockout embryos (Figures 2A,F). Importantly, we found that these abnormal phenotypes of motor neurons could not recover at later stages we checked (Supplementary Figure 3).

Moreover, the morphology of motor neurons was significantly affected in the *insm1a* mutants (Figures 2A,D,E). The axons of Caps in *insm1a* mutants were shorter and failed to reach the ventral musculatures. The branches density of the Caps in *insm1a* mutants was higher than that in control. For example, in the *insm1a* mutants, there were around 34 branch points of per 1 mm CaP axon at 48 hpf, while only 31 in control embryos (Figure 2E). With the larvae development, the excess branching became more, and more pronounced (Supplementary Figure 3). In addition, statistical analysis revealed that the average length of each CaP anon in the *insm1a* mutants was 707.9 μm at 48 hpf, while in control embryos it increased to 1367.9 μm (Figure 2D). Interestingly, we also found that the distances between adjacent CaPs were significantly variant in *insm1a* mutants (Figure 2C).

In order to validate the developmental defects of motor neuron was specifically caused by the *insm1a* inactivation, further experiments were carried out. The embryos that injected with an *insm1a* translation blocking morpholino displayed the similar motor neuron with that observed in the *insm1a* mutants (Supplementary Figure 4). To confirm phenotypic specificity induced by the *insm1a* MO injection, we performed rescue experiment by co-injection of 50 ng of *insm1a* mRNA with *insm1a* MO into zebrafish embryos, and the results showed that the co-injection significantly decreased the loss, and premature branching of PMNs (Supplementary Figure 4E). Taken together, these results indicated those motor neuron developmental defects were caused by loss of *insm1a*.

### Insm1a deficiency suppressed neuronal cells differentiation

**Figure 3:**
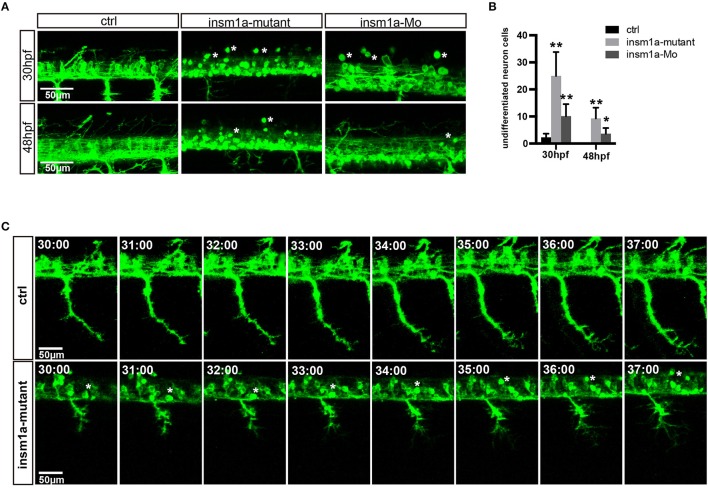
*Insm1a* deficiency suppressed neuronal cells differentiation. **(A)** Confocal imaging analysis of primary motor neuron in control group, *insm1a* mutant group and morphant group at 30 and 48 hpf *Tg(mnx1:GFP)*^*ml2*^. Phenotypes of neuronal cells in the spinal cord in control group, morphant group, and insm1a mutant groups at 30 hpf and 48 hpf. Asterisks indicate undifferentiated neuronal cells. Scale bar = 50 μm. **(B)** Quantification of the undifferentiated neuronal cell in the *insm1a* different treatment zebrafish. Asterisks above the bars indicate significant differences (^*^P < 0.05, ^**^P < 0.01). **(C)** Time-lapse imaging analysis of the primary motor neuron in control group and insm1a mutant groups. Asterisks represent undifferentiated neuronal cells. Scale bar = 50 μm

The confocal imaging analysis discovered that there were a number of round and not well-differentiated GFP positive cells in *Tg(mnx1:GFP)*^*ml*2^ *insm1a* mutants (Figure 3A). Statistical analysis showed that at 30 and 48 hpf the number of these undifferentiated cell in the *insm1a* deficiency zebrafish was significantly higher than that in the control fish (Figure 3B). We also observed these undifferentiated cells in *insm1a* morphants, however the number was less than that in mutants (Figures 3A,B).

To further investigate the cellular mechanism underlying the motor neuronal defects in *insm1a* deficient embryos, we performed confocal time-lapse imaging analysis. It was found that in control embryos the axon of CaP sprouted from the spinal cord, and extended toward to the ventral muscle (Figure 3C). In control embryos the axon of CaP started to branch when it passed through the midline, while in *insm1a* mutants the axon initiated to branch once it came out from the spinal cord (Figures 3A,C, Supplementary Movies 1, 2). In addition, we found that those round GFP positive cells did not develop neuronal projections (Figures 3A,C, Supplementary Movie 1).

### Knockout of *insm1a* reduced the zebrafish swimming activity

**Figure 4:**
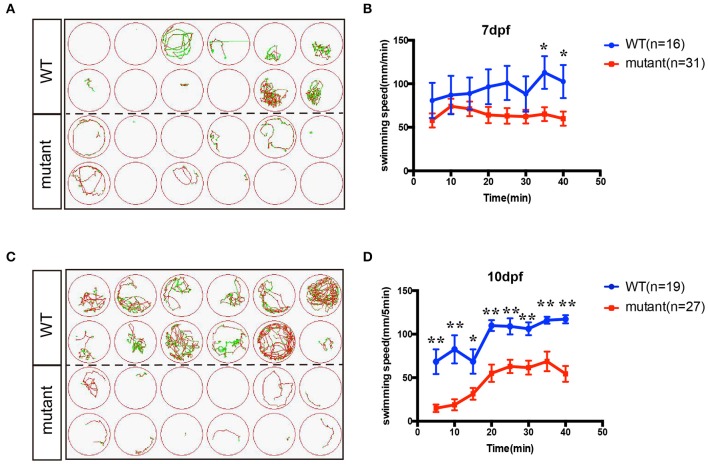
The swimming behavior analysis of control and *insm1a* mutant zebrafish embryos at 7 and 10 dpf. **(A,C)**. The swimming trajectory of the control and *insm1a* mutant zebrafish embryos at 7 and 10 dpf. **(B,D)**. Quantification of the swimming distance of control and *insm1a* mutant zebrafish embryos at 7 and 10 dpf per 5 mins (*n* = 36 in each group). Asterisks indicate the significant difference (^*^P < 0.05, ^**^P < 0.01).

In order to investigate whether the motor neuron defects affects the motor ability, *insm1a* mutant zebrafish larvae were further performed for 40-min free-swimming activity test independent of any stimuli at 7 and 10 dpf. It demonstrated that the movement trajectory and swimming distance per 5 mins, which could reflect the swimming speed, of *insm1a* mutant zebrafish larvae were significantly decreased compared to that in the control (Figure 4). The movie in the Supplementary Material showed that swimming behavior could be easily discovered in the control group, while the zebrafish in mutant group kept involuntomotory (Supplementary Movie 3). Additionally, we also discovered that under the stereoscopic microscope the mutant zebrafish became insensitive to the touch stimuli (data not shown).

### The *insm1a* deficiency caused alteration of gene expression involved in motor neuron development

**Figure 5:**
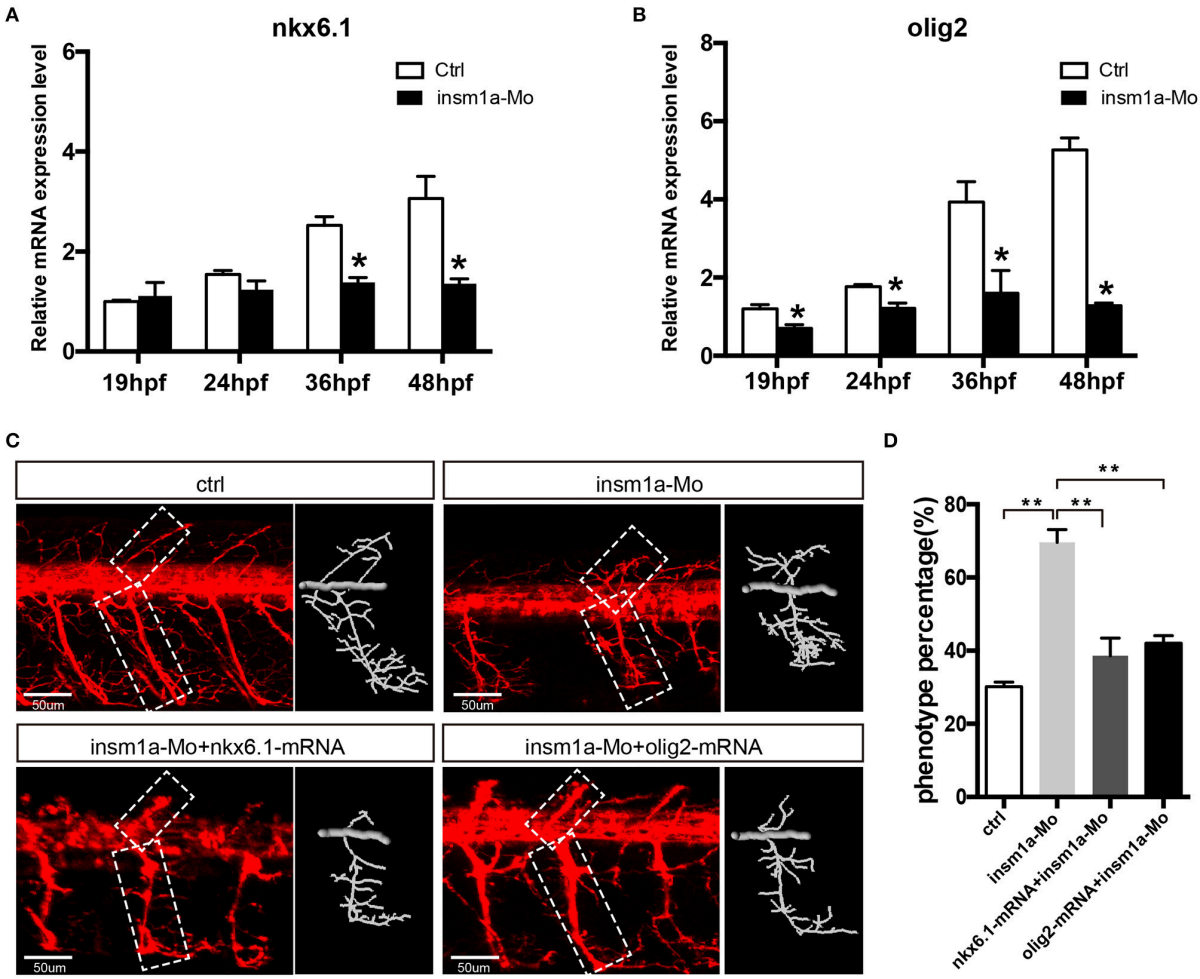
Over expressions of *nkx6.1* and *olig2* rescued the motor neuron defects in *insm1a* deficient embryos. **(A,B)**. Effects of *insm1a* knockdown on the expressions of *nkx6.1* and *olig2* at 19, 24, 36, and 48 hpf. Asterisks indicate significant differences (^*^P < 0.05). **(C)** Abnormal Caps in insm1a knockdown zebrafish embryos were restored by co-injection of *nkx6.1* or *olig2* mRNA. Diagrams of Caps in dash line are displayed near the corresponding confocal image. Scale bar = 50 μm. **(D)** Quantification of zebrafish embryos with abnormal Cap primary motor neuron. Asterisks indicate significant differences (^**^P < 0.01).

Since insm1a is a transcription factor, we supposed that motor neuron developmental defects in *insm1a* deficient embryos were associated with altered expression of downstream genes of *insm1a* or the genes participating in the motor neuron development. Based on the previous studies, *NNR2a, NNR2b, islet2, Asci1a, Asci1b, shh, Ngn2, Nkx6.1*, and *olig2* were selected to do the qRT-PCR analysis in wild-type (WT) and *insm1a* deficiency zebrafish embryos (Park et al., 2002; Hutchinson et al., 2007; Davis-Dusenbery et al., 2014; Barreiro-Iglesias et al., 2015). The results showed that expressions of *NNR2a, NNR2b, islet2, Asci1a*, and *Asci1b* were significantly influenced in the *insm1a* deficiency zebrafish compared to the control (Supplementary Figure 5). We also found that the expression of *shh* was obviously elevated in *insm1a* mutants at 19, 24, and 36 hpf (Supplementary Figure 5). Interestingly, *olig2* and *nkx6.1* transcripts dramatically decreased in *insm1a* deficient embryos (Figures 5A,B).

### *Olig2* and *nkx6.1* over expression rescued the motor neuron defects in *insm1a* deficient embryos

As the downregulation of *olig2* and *nkx6.1* in *insm1a* loss of function embryos, we reasoned that insm1a might bind the transcriptional regulatory elements of these two genes. Based on the JASPAR 2016 database (Mathelier et al., 2016) analysis, we found that both *olig2* and *nkx6.1* contained the putative binding sites of Insm1a, suggesting Insm1a directly regulates the expression of *olig2*, and *nkx6.1* during PMNs development. To investigate whether the motor neuronal defects in *insm1a* deficient embryos were caused by reduced expression of *olig2* and *nkx6.1*, we tried to rescue the phenotype with olig2 and nkx6.1 gain of function in *insm1a* deficient embryos. It was shown that co-injection both *olig2 and nkx6.1* mRNA respectively with *insm1a* MO significantly reduced the motor neuronal defects caused by loss of *insm1a* (Figures 5C,D). 69.6% zebrafish embryos injected with *insm1a* MO at 48 hpf had the motor neuron developmental defects, while only 42.1% had the motor neuronal phenotype in the *olig2* mRNA and *insm1a* MO co-injection group (Figure 5D). Similarly, after *nkx6.1* mRNA and *insm1a* MO co-injection, the ratio of motor neuronal phenotype decreased to 38.6% (Figure 5D).

## Discussion

As one of the most conserved zinc-finger transcriptional factor, insm1a plays important roles in various biological processes in vertebrates (Wildner et al., 2008; Jacob et al., 2009; Forbes-Osborne et al., 2013; Osipovich et al., 2014; Jia et al., 2015b; Lorenzen et al., 2015). Previous studies have identified its role in regulating the endocrine cells divisions of the pancreas, the neuroendocrine development, the differentiation of retina progenitors and neurogenesis of nervous system (Gierl et al., 2006; Duggan et al., 2008; Farkas et al., 2008; Jacob et al., 2009; Lan and Breslin, 2009; Ramachandran et al., 2012). Currently, our data in this study provided with new insights into the role of insm1a in motor neuron development.

Our WISH data and previous study (Ramachandran et al., 2012) demonstrated that *insm1a* transcripts were detected in retina and spinal cord at 24 hpf. Furthermore, imaging analysis of our established transgenic reporter line *Tg(insm1a: EGFP)*^*ntu804*^ *and Tg(insm1a: mCherry)*^*ntu805*^ verified the results of *in situ* hybridization, and revealed the expression of EGFP or mCherry that were driven by *insm1a* promoter in the PMNs. It is well-known that the spinal cord contains PMNs which project their axons out of the spinal cord to the terminal musculature with the embryo development (Davis-Dusenbery et al., 2014). Taken together, the localization data of *insm1a* from both *in situ* hybridization analysis and the study based on transgenic reporter line suggested that *insm1a* might participate in the regulation of PMNs development.

To test whether *insm1a* was required for formation of PMNs, we generated CRISPR/Cas9-mediated *insm1a* mutants, and showed that obvious motor neuron loss and defects of the PMNs axons. Moreover, we also performed *insm1a* knockdown, and the results showed similar PMNs defects as the ones produced by the *insm1a* knockout. In wild embryos, exuberant side branches developed at around 72 hpf, and then invaded into myotome to form distributed neuromuscular synapses (Liu and Westerfield, 1990; Downes and Granato, 2004). These results suggested that *insm1a* was pivotal for the primary motor axon development to block precociously extending into muscle territories. Additionally, locomotion analysis displayed a typical low activity swimming behavior in *insm1a* mutant zebrafish. It was known motor neuron was a major kind of cell type that regulated swimming behavior in zebrafish during early development (Brustein et al., 2003). Previous studies also showed a significant involvement of motor neuron in the overall locomotor behavior (Flanagan-Steet et al., 2005; Levin et al., 2009). Currently, the decrease of swimming activity in this study was consistent with the motor neuron defects in the *insm1a* knockout zebrafish.

Another prominent phenotype in the *insm1a* deficiency zebrafish was the disorganized distance between adjacent Caps, which might be caused by the ectopic departure of motor axons from the spinal cord (Palaisa and Granato, 2007). During the zebrafish PMNs development, the three kinds of PMN axons firstly longitudinally migrated toward a segmental spinal cord exit point, and then diverged to individual-specific trajectories (Eisen et al., 1986; Myers et al., 1986). It has been reported that axonal exit sites at the spinal cord might be restricted and conserved (Niederlander and Lumsden, 1996). However, the change of distance between adjacent motor axon and the formation of abnormal axons in our study suggested that the motor axons could form exit points at any positions along entire length of spinal cord in *insm1a* mutants. The similar phenotypes were also showed in *plexin A3* and *semaphorin 3A* morphants (Feldner et al., 2007; Palaisa and Granato, 2007; Tanaka et al., 2007). Additionally, Birely et al. reported the phenotype that motor axons departed from the spinal cord at the ectopic points accompanied with defects in slow muscle fiber development (Birely et al., 2005). These studies suggested that the low activity swimming behavior in *insm1a* mutant zebrafish might be involved in the ectopic departure of motor axons from the spinal cord.

In this study we also found that loss function of Insm1a obviously impaired the motor neuronal differentiation. Similarly, It was shown that Insm1a regulated cell differentiation and migration in zebrafish retinal development and regeneration (Ramachandran et al., 2012; Forbes-Osborne et al., 2013). In vertebrates, Insm1 stimulates cell cycle exit by suppressing expression of cell proliferation related genes and relieving repression of p57kip2, a cyclin kinase inhibitor that along with p27kip1 drives cell cycle exit (Dyer and Cepko, 2001). One consequence of insm1a driven cell cycle exit is progenitor differentiation (Ramachandran et al., 2012). The undifferentiated cells in the spinal cord of *insm1a* mutants confirmed the role of this transcriptional factor in cell differentiation in more cell types.

A series of genes have been identified to contribute to motor neuron formation and development (Park et al., 2002; Cheesman et al., 2004). It has been reported that *nkx6.1* and *olig2* were dynamically expressed in zebrafih motor neuron and required for motor neuron development. Downregulation of the two genes lead to developmental defect of motor neuron, which was similar with that in *insm1a* mutant (Park et al., 2002; Cheesman et al., 2004; Hutchinson et al., 2007). Conversely, overexpression of *nkx6.1* or *olig2* by mRNA injection could significantly promote the development of the PMNs (Park et al., 2002; Hutchinson et al., 2007). Current study revealed that the inactivation of *insm1a* resulted in the significant decrease of *nkx6.1* and *olig2* expression levels. Furthermore, *olig2*, and *nkx6.1* overexpression rescued the motor neuron defects in *insm1a* deficient embryos. These data suggested that *insm1a* regulated the motor neuron development, at least in part, by regulating the expressions of *olig2*, and *nkx6.1*.

## Ethics statement

All animal experimentation was carried out in accordance with the NIH Guidelines for the care and use of laboratory animals (http://oacu.od.nih.gov/regs/index.htm) and ethically approved by the Administration Committee of Experimental Animals, Jiangsu Province, China [Approval ID: SYXK (SU) 2007–0021].

## Author contributions

DL, RC, and QSZ conceived the project. JG, XW, CZ, XNW, XCD, FQ, YS, and YG performed most of the experiments. XHD and QXZ generated the mutants. DL, JG, and XW analyzed the data and prepared the manuscript. All authors commented and approved the manuscript.

### Conflict of interest statement

The authors declare that the research was conducted in the absence of any commercial or financial relationships that could be construed as a potential conflict of interest.
