# Supplementary material for: Insm1a Regulates Motor Neuron Development in Zebrafish
Source: Front Mol Neurosci. 2017 Aug 28;10:274. doi: 10.3389/fnmol.2017.00274 (PMC5581358; doi:10.3389/fnmol.2017.00274)
Supplement: Supplementary file 1 [file Table1.docx]

| **Supplementary Table 1 Primers and oligos are used in this study.** | |
| --- | --- |
| **Primer name** | **Sequence** (5’-3’) |
| insm1a-test-F | CTCTGATTTCAACCCGACTG |
| insm1a-test-R | TTGCGTAAAGCGTTCTCACT |
| Insm1a-probe-L | ACAAATGCTCCAGGATCGTC |
| Insm1a-probe-R | GGTGGCATTTGTTGATGTGT |
| Insm1a-promoter-L | ATTTCCTCTTTTTCGCAGTTATTTT |
| Insm1a-promoter-R | CAGAGGCACACCTGGTACTATATTT |
| Insm1a-sgRNA-F | TAATACGACTCACTATAggaaccccgagacagtctaGTTTTAGAGCTAGAAATAGC |
| olig2 F1 | CACCTGCTACCGGAATATCGA |
| olig2 R1 | AAACCCACGGACTTCTTGACG |
| Nkx6.1 F1 | TTACCCAGCCTACCCGTTATCT |
| Nkx6.1 R1 | GAGGATGTCGTTTATTCCGTGA |
| MNR2a F1 | GCTGCGTGCGGGACTCATAAT |
| MNR2a R1 | CTGGTGAAGGCTGTGCGTGGT |
| MNR2b F1 | CCTCAGTCTGGTTTGATGGGTA |
| MNR2b R1 | CGTTTGGGTCTGGAAAGGTATT |
| Ngn2 F1 | TTACATTTGGGCGTTGTCAGA |
| Ngn2 R1 | TTTGTGGAGTGCCACTTGGAG |
| Islet2 F1 | GTCCGAGACGGCAAGACTTATT |
| Islet2 R1 | CCGAGCACCTAAAACACTCCAT |
| Shha F1 | TATTACGAGTCCAAAGCCCACA |
| Shha R1 | TGATGAAGTCGCTGAACACCAG |
| YAscl1b F | CCTTCAACGGACTGGGCTACAC |
| YAscl1b R | CAGCGTCTCCACTTTGCTCATCT |
| YAscl1a F | GGACGAGCATGACGCTGTAAG |
| YAscl1a R | CAGTTGGTGAAGTCCAGGAGC |
| YEF1a-F1 | TGATCTACAAATGCGGTGGA |
| YEF1a-R1 | CAATGGTGATACCACGCTCA |
| insm1a-mRNA-BamH1-F | CGGGATCCCGCTGAAGTGCCTTTCAGCTG |
| insm1a-mRNA-EcoR1-R | GGAATTCC CACAGGTTGTCTTCAGCAGG |
| olig2-mRNA- EcoR1-F | GGAATTCCGAGAAACTGAGAGCGCACT |
| olig2-mRNA-Xba1-R | GCTCTAGAGCAGTCTGTGGTGGCTTCTCA |
| Nkx6.1-mRNA- EcoR1-F | GGAATTCCTGGTTATGTTAGCGGTGGGG |
| Nkx6.1-mRNA-Xba1-R | GCTCTAGAGCTCTGACAACCCATTTCCCGT |
