# Supplementary material for: Insm1a Regulates Motor Neuron Development in Zebrafish
Source: Front Mol Neurosci. 2017 Aug 28;10:274. doi: 10.3389/fnmol.2017.00274 (PMC5581358; doi:10.3389/fnmol.2017.00274)
Supplement: Supplementary file 5 [file DataSheet1.DOCX]

**Supplementary Materials**

**Title: Insm1a regulates motor neuron development in zebrafish**

Jie Gong^1, *^, Xin Wang^2, *^, Chenwen Zhu^2^, Xiaohua Dong^3^, Qinxin Zhang^3^, Xiaoning Wang^2^, Xuchu Duan^1^, Fuping Qian^4^, Yu Gao^2^, Qingshun Zhao^3, #^, Renjie Chai^4, 2, #^, Dong Liu^2, #^

1 School of life science, Nantong University, Nantong, China

2 Co-innovation Center of Neuroregeneration, Key Laboratory of Neuroregeneration of Jiangsu and Ministry of Education, Nantong University, Nantong, China

3 MOE Key Laboratory of Model Animal for Disease Study, Model Animal Research Center, Nanjing University, Nanjing, China

4 Key Laboratory for Developmental Genes and Human Disease, Ministry of Education, Institute of Life Sciences, Southeast University, Nanjing, China

*, these authors contributed equally to this work

#, authors for correspondence


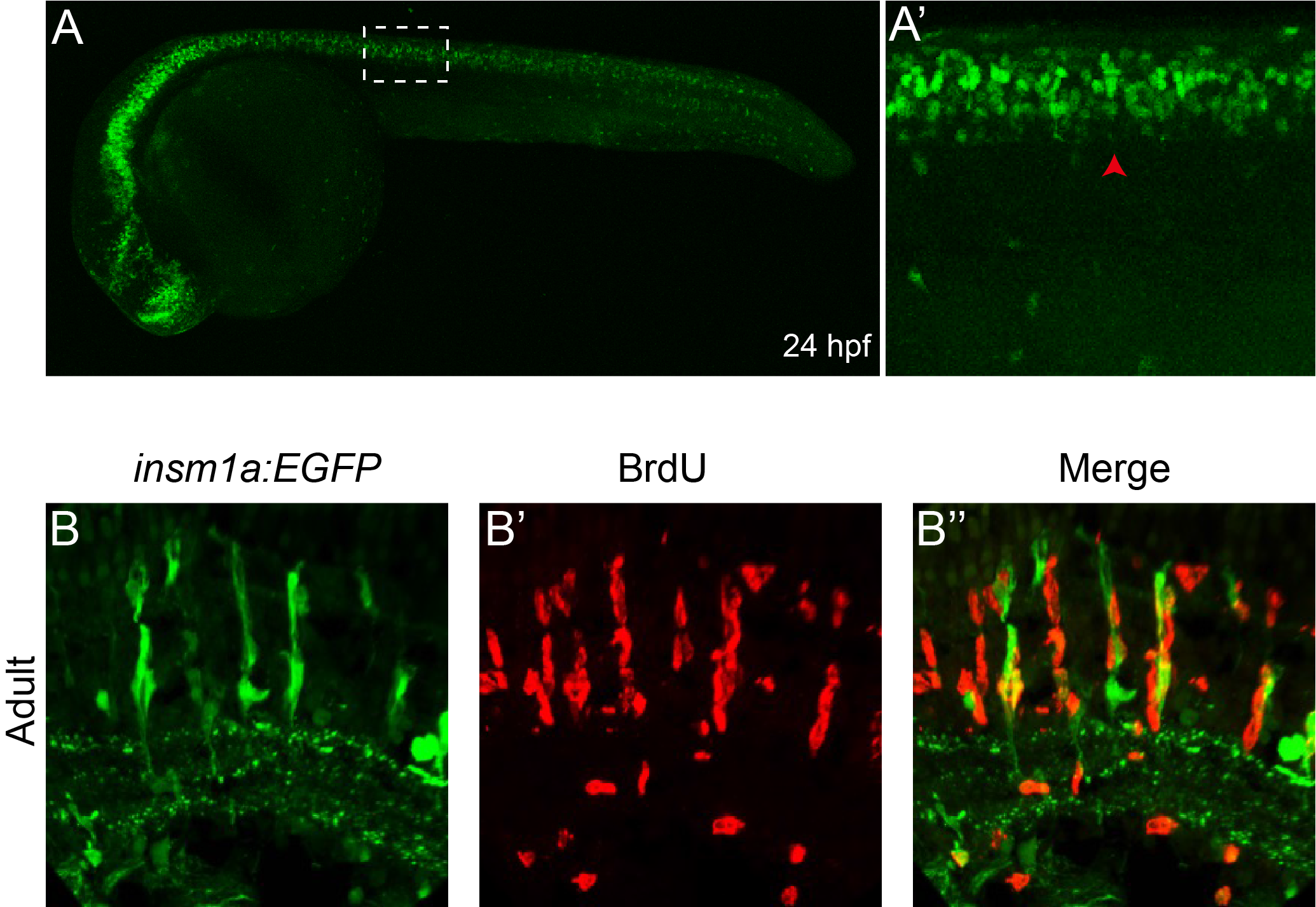


**Supplementary Figure 1. *Insm1a:GFP* transgene expression patterns in embryonic and adult zebrafish.** A, Confocal imaging analysis of *Tg(insm1a: EGFP) ^ntu804^* transgenic zebrafish line at 24 hpf. A’ The magnified image of rectangle in dash line. B-B’’ Confocal imaging analysis of injured retina of adult *Tg(insm1a: EGFP) ^ntu804^* transgenic zebrafish line.


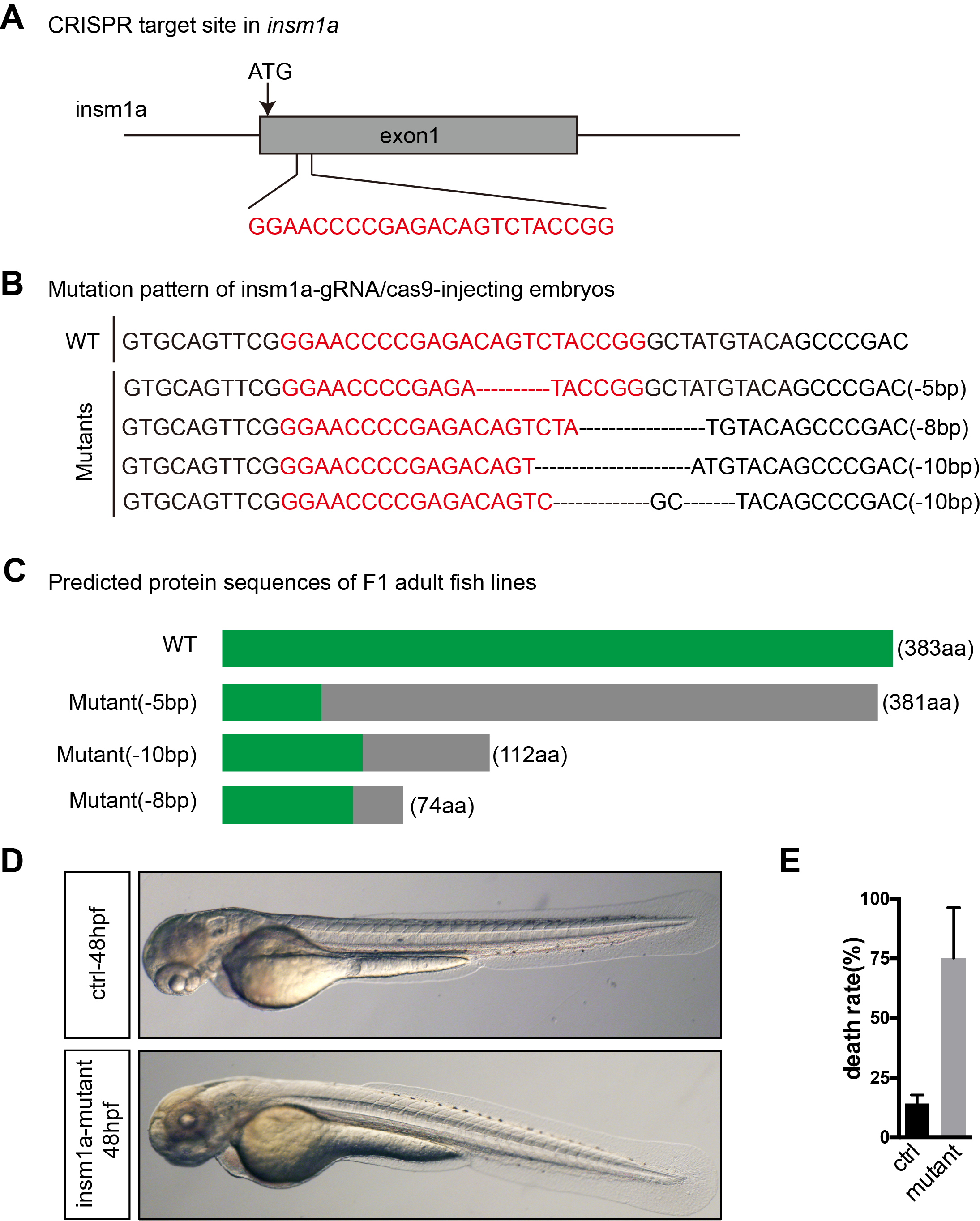


**Supplementary Figure 2. Generation of zebrafish insm1a mutant using CRISPR/Cas9 system.** A. Schematic diagram showing the targeting site of the sgRNA on the first exon of *insm1a* gene. Starting codon (ATG) site is indicated by arrow. B. Mutation pattern of *insm1a*-gRNA/cas9-injecting embryos. Numbers in the brackets show the numbers of nucleotides were deleted (−). C. Schematic diagram showing the predicted proteins encoded by the three mutated alleles. The mutants are reading frameshift mutations that result in truncated proteins. The gray rectangles indicate the wrong coded amino acid sequences. D. The imaging analysis of *insm1a* mutant and wide type zebrafish at 48 hpf in bright field. E. Death rate of zebrafish embryos in control group and *insm1a* mutant groups at 15dpf.

**
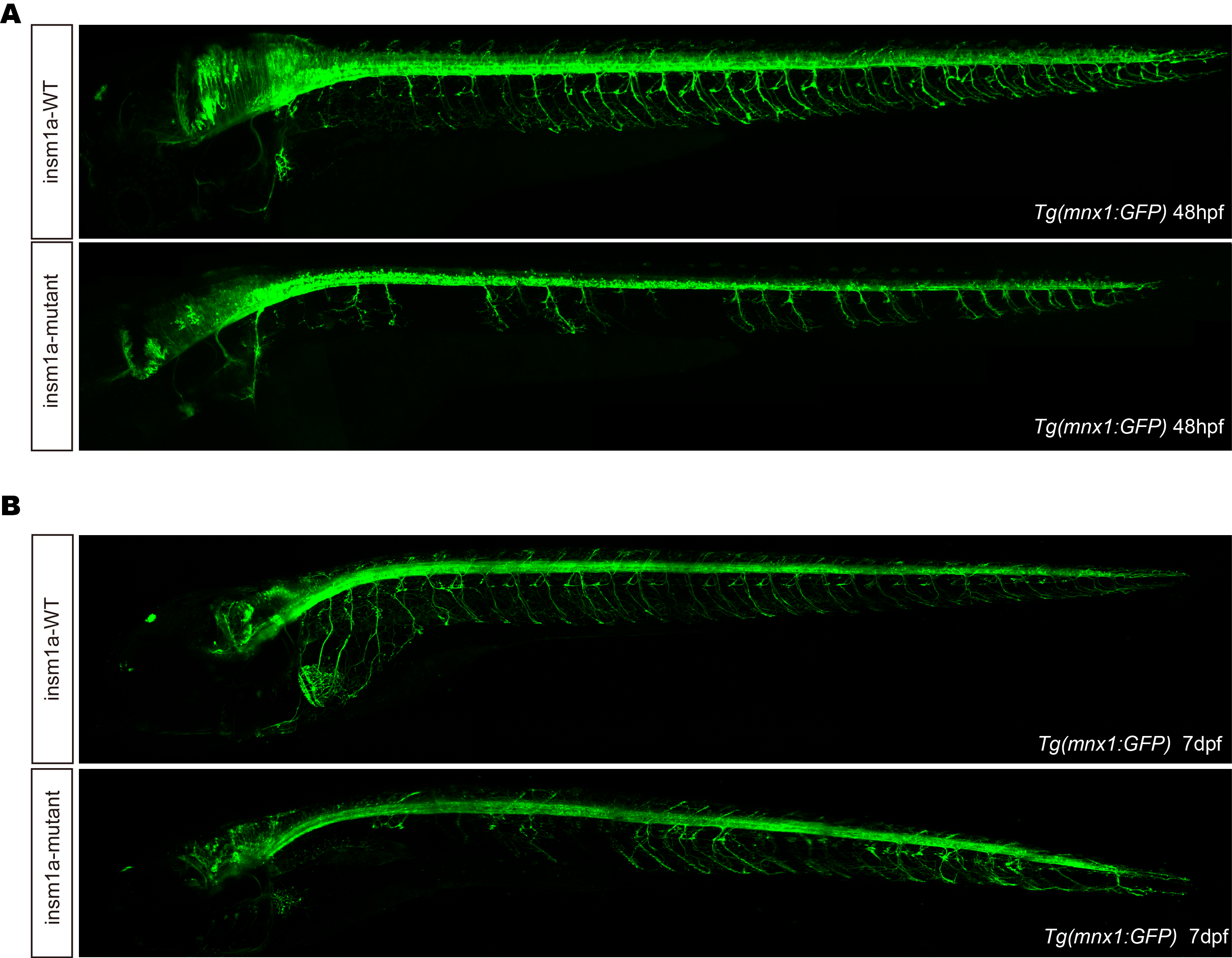
**

**Supplementary Figure 3. Confocal imaging analysis of of *insm1a* mutant *Tg(mnx1:GFP)^ml2^* transgenic zebrafish at 48 hpf and 7 dpf.**

**
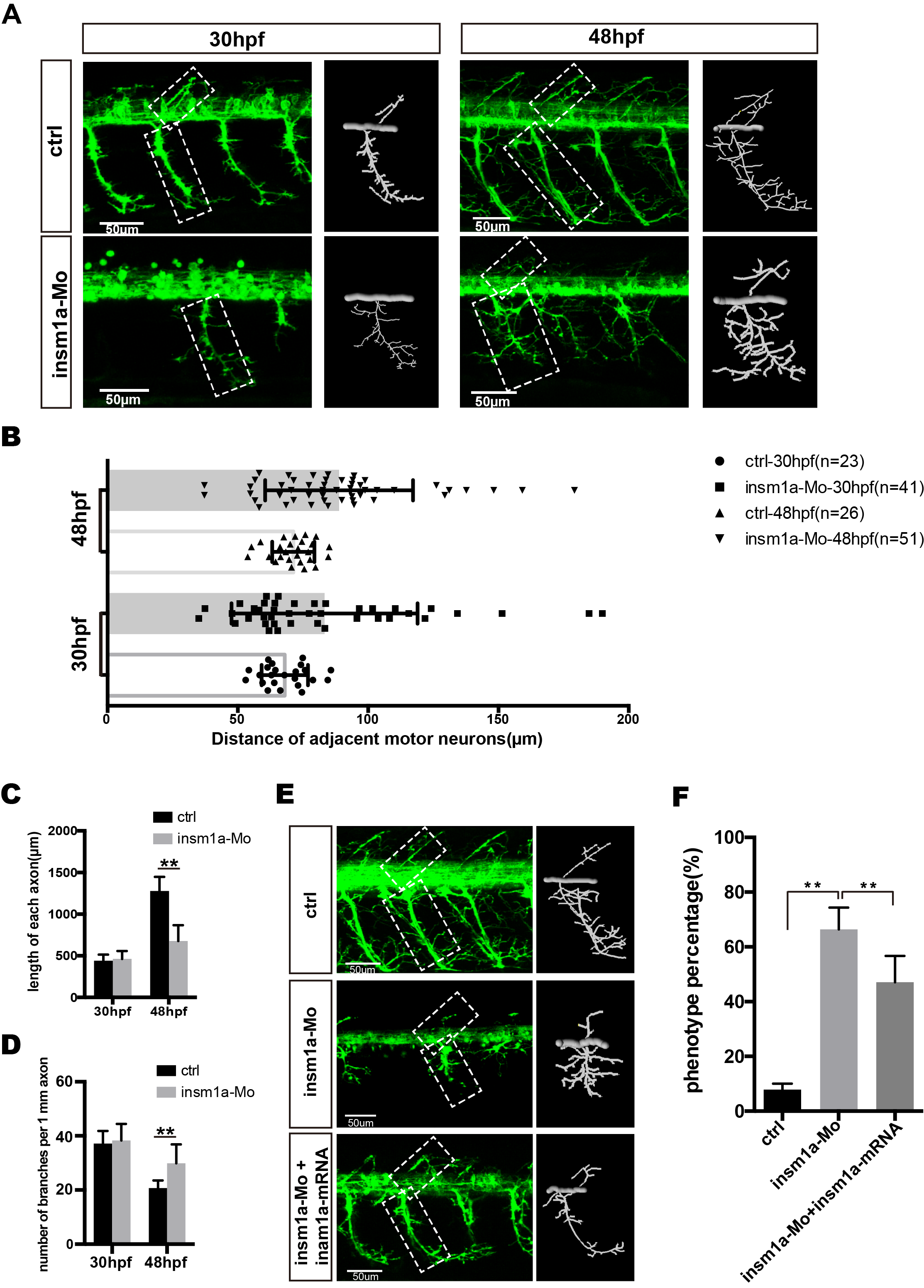
**

**Supplementary Figure 4. Primary** **motor neuron developmental defects in the *insm1a* morphants.** A. Confocal imaging analysis of primary motor neuron in control group and *insm1a* morpholino injected groups at 30 hpf and 48 hpf *Tg(mnx1:GFP)^ml2^*. Caps in dash line are showed in diagrams. Scale bar = 50 μ m. B. Quantification of distance between adjacent motor neurons (μm) in control group and insm1a morpholino injection groups at 30 hpf (n=23 and 41 respectively) and 48 hpf (n=26 and 51 respectively). C and D. The length and branching number of Cap axons in control group and *insm1a* morphant groups at 30 hpf and 48 hpf. Asterisks above the bars are significantly different (P<0.05). Values with ** above the bars are significantly different (P<0.05). E. Abnormal Caps in *insm1a* knockdown zebrafish embryos were restored by co-injection of *insm1a* mRNA with the morpholino. F. Quantification of zebrafish embryos with abnormal Caps.

**
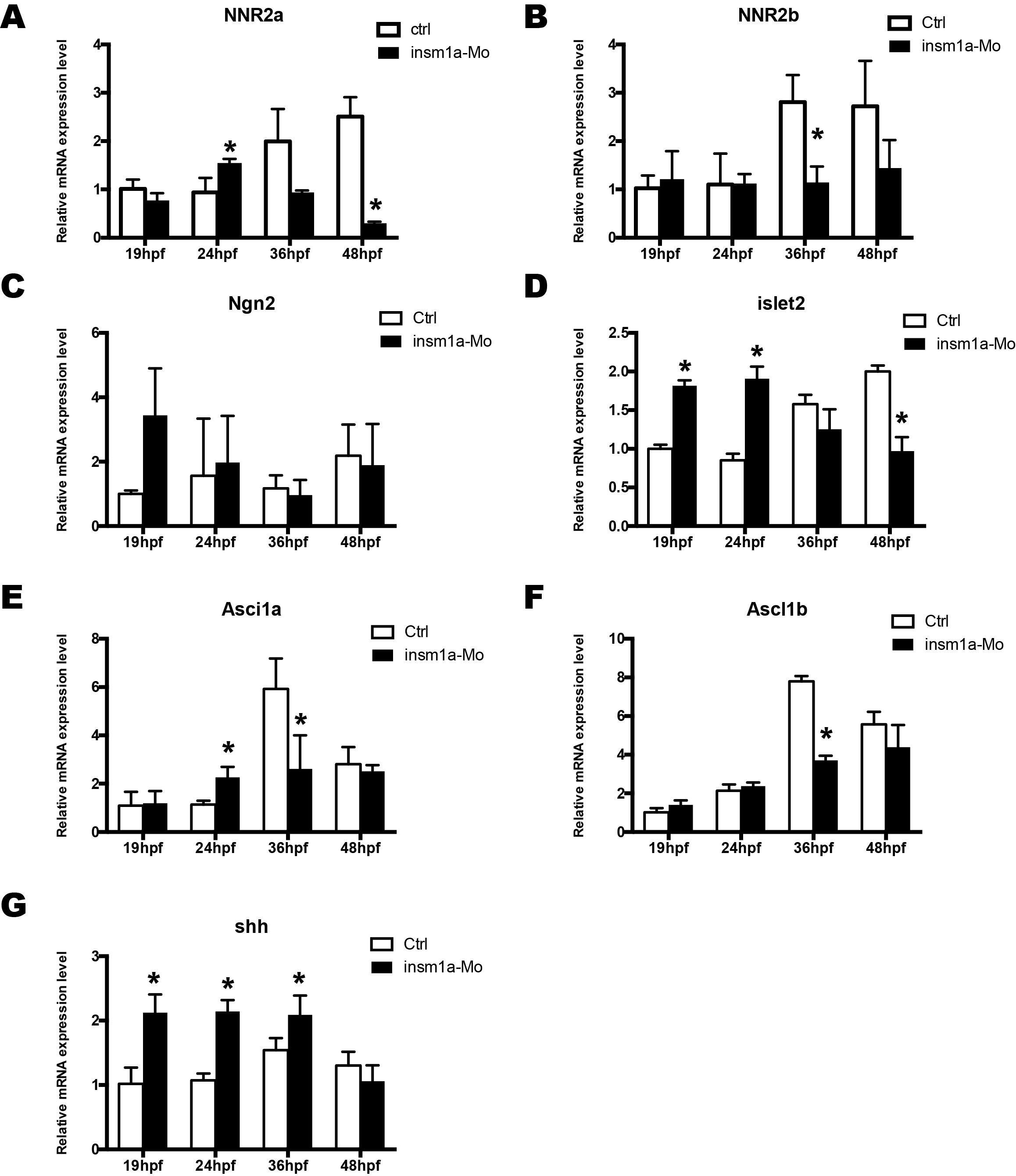
**

**Supplementary Figure 5. Effects of *insm1a* morpholino injection on the expressions of motor neuron regualtion related genes.** Experimental embroys were sampled at 19, 24, 36 and 48 hpf. Asterisks indicate significant differences compared with the control (P<0.05).

**Supplementary movie 1. Time-lapse microscopy analysis of the motor neuron development in control zebrafish embryos.**

**Supplementary movie 2. Time-lapse microscopy analysis of the motor neuron development in *insm1a* mutant zebrafish embryos.**

**Supplementary movie 3. Individual swimming movie frames of the control and *insm1a* mutant 7 dpf zebrafish. The control embryos are contained in the top two rows. The *insm1a* mutant zebrafish embryos are contained in the bottom two rows.**
